# An algorithm for dementia diagnosis

**DOI:** 10.1002/alz70857_102930

**Published:** 2025-12-25

**Authors:** Howard Chertkow

**Affiliations:** ^1^ Rotman Research Institute, Baycrest Health Sciences, Toronto, ON, Canada

## Abstract

**Background:**

Recent work (Jack et al, 2024) proposes an AD diagnostic framework which moves biomarkers to the centre, proposing that short of biomarker access, clinicians should simply refer to patients as having “dementia” or “MCI” without further differentiation. Such a move would have a significant negative impact on all patients and physicians lacking access to diagnostic biomarkers, ignores their clinical acumen, and negates the considerable progress in clinical neurology over the past generation.

**Method:**

A review of the literature on clinical diagnosis was supplemented by database review of convenience sample of 52 subjects with clinical probable AD and known amyloid status (csf or PET), as well as 145 subjects from the Canadian COMPASS‐ND dataset with known amyloid status (csf).

**Result:**

A proposal is made to continue diagnostic algorithms promoting distinction of dementia patients into those with and without motor features. A proposal is further made to refer to clinical “AD phenotype” dementia patients without motor features as having “Alzheimer Syndrome”. A further level of diagnosis would consist of distinguishing Alzheimer Syndrome patients into those with and without presence of amyloid. This can be accomplished with about 80% accuracy using clinical guidelines such as CAPS (A Clinical beta Amyloid Positivity prediction Score; Lahiri et al, 2024) utilizing presence of neuropsychiatric features, rapidity of cognitive decline, and presence of white matter disease. Greater accuracy than this would require use of amyloid biomarkers.

**Conclusion:**

Changes in terminology are now necessary to acknowledge the language proposed by the academic community which has easy access to biomarkers, without disempowering clinicians (most of them) who lack easy access to the biomarkers. Good (but not excellent) level of specifying likelihood of amyloid being present, can likely be achieved with more accessible clinical markers. This approach should be pursued and replicated among diverse populations of patients around the world.

